# Cardiolaminopathies from bench to bedside: challenges in clinical decision-making with focus on arrhythmia-related outcomes

**DOI:** 10.1080/19491034.2018.1506680

**Published:** 2018-10-18

**Authors:** Giuseppe Boriani, Elena Biagini, Matteo Ziacchi, Vincenzo Livio Malavasi, Marco Vitolo, Marisa Talarico, Erminio Mauro, Giulia Gorlato, Giovanna Lattanzi

**Affiliations:** a Cardiology Division, Department of Biomedical, Metabolic and Neural Sciences, University of Modena and Reggio Emilia, Policlinico di Modena, Modena, Italy; b Institute of Cardiology, Department of Experimental, Diagnostic and Specialty Medicine, University of Bologna, S.Orsola-Malpighi University Hospital, Bologna, Italy; c CNR Institute of Molecular Genetics, Unit of Bologna, Bologna, Italy; d Laboratory of Musculoskeletal Cell Biology, Rizzoli Orthopedic Institute, Bologna, Italy

**Keywords:** Arrhythmia, emerin, Emery-Dreifuss muscular dystrophy, heart failure, lamin A/C, sudden cardiac death, thromboembolism, stroke

## Abstract

Lamin A/C gene mutations can be associated with cardiac diseases, usually referred to as ‘cardiolaminopathies’ characterized by arrhythmic disorders and/or left ventricular or biventricular dysfunction up to an overt picture of heart failure. The phenotypic cardiac manifestations of laminopathies are frequently mixed in complex clinical patterns and specifically may include bradyarrhythmias (sinus node disease or atrioventricular blocks), atrial arrhythmias (atrial fibrillation, atrial flutter, atrial standstill), ventricular tachyarrhythmias and heart failure of variable degrees of severity. Family history, physical examination, laboratory findings (specifically serum creatine phosphokinase values) and ECG findings are often important ‘red flags’ in diagnosing a ‘cardiolaminopathy’. *S*udden arrhythmic death, thromboembolic events or stroke and severe heart failure requiring heart transplantation are the most dramatic complications of the evolution of cardiolaminopathies and appropriate risk stratification is clinically needed combined with clinical follow-up. Treatment with cardiac electrical implantable devices is indicated in case of bradyarrhythmias (implant of a device with pacemaker functions), risk of life-threatening ventricular tachyarrhythmias (implant of an ICD) or in case of heart failure with wide QRS interval (implant of a device for cardiac resynchronization). New technologies introduced in the last 5 years can help physicians to reduce device-related complications, thanks to the extension of device longevity and availability of leadless pacemakers or defibrillators, to be implanted in appropriately selected patients. An improved knowledge of the complex pathophysiological pathways involved in cardiolaminopathies and in the determinants of their progression to more severe forms will help to improve clinical management and to better target pharmacological and non-pharmacological treatments.

## Laminopathies and the heart

Lamins A and C are intermediate filament nuclear envelope proteins supporting the structure and function of the nucleus of eukaryotic cells and are encoded by the *LMNA* gene [1–3].

Lamin A/C gene mutations can be associated with cardiac diseases, usually referred to as ‘cardiolaminopathies’ characterized by arrhythmic disorders and/or left ventricular or biventricular dysfunction up to an overt picture of heart failure.

The first report stressing the importance of lamin A/C mutations as the genetic basis for important cardiac consequences in terms of dilated cardiomyopathy and arrhythmic events was the study by Bonne et al. on Emery-Dreifuss muscular dystrophy [1].

**Figure 1.**
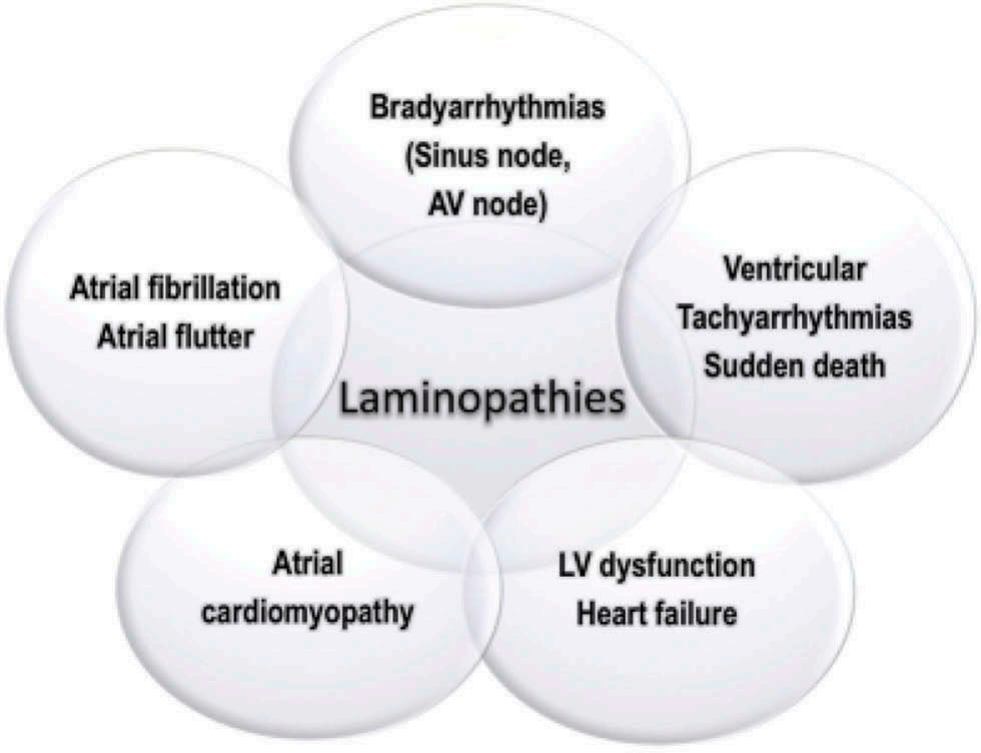
Spectrum of cardiac involvement in cardiolaminopathies, with regard to arrhythmic disturbances and heart failure. Legend: AV: atrio-ventricular, LV: left ventricular.

In Emery-Dreifuss muscular dystrophy [1], cardiac abnormalities, leading to life-threatening bradyarrhythmias either of sinus and atrial origin (sino-atrial blocks, atrial standstill) or atrioventricular blocks (up to third degree atrioventricular block), ventricular tachyarrhythmias, sudden death and dilated cardiomyopathy with heart failure of variable degree are not strictly correlated with the severity of the neuromuscular disease affecting the skeletal muscles [4]. Figure 1 shows the phenotypic cardiac manifestations of laminopathies, which are frequently mixed in complex clinical patterns. Family history, physical examination, laboratory findings (specifically serum creatine phosphokinase values) and ECG findings are often important ‘red flags’ in diagnosing a ‘cardiolaminopathy’.

Laminopathies involve a wide spectrum of diseases affecting striated muscle, the heart, the adipose tissue, and peripheral nerves or can be a multi-organ and multi-system disease as in the Hutchinson-Gilford progeria syndrome [3,5] and cardiac disturbances may be present in patients with quite different phenotypes due to lamin A/C mutations.

Phenotypic penetrance is age-related but the expression is extremely heterogeneous, so that muscular and arrhythmic disease can be present in combination in the same patient, or one phenotypic manifestation can appear earlier than the other or even not become overt for a long time [6]. Moreover, both the severity of the disease and its progression may have a marked interindividual variability also within the same family of affected patients.

From a cardiological point of view, characterization of a patient as affected by a laminopathy with heart involvement (‘cardiolaminopathy’) is of crucial importance, since clinical and prognostic implications, as well as specific management strategies, can be different, particularly with regard to prevention of sudden cardiac death. A specific diagnosis, based on genetic testing, and appropriate risk stratification with referral to expert centers involving a multidisciplinary team for appropriate decision-making is currently needed in patients affected by the various manifestations of this disease.

## Pathogenetic pathways in cardiolaminopathies

**Figure 2.**
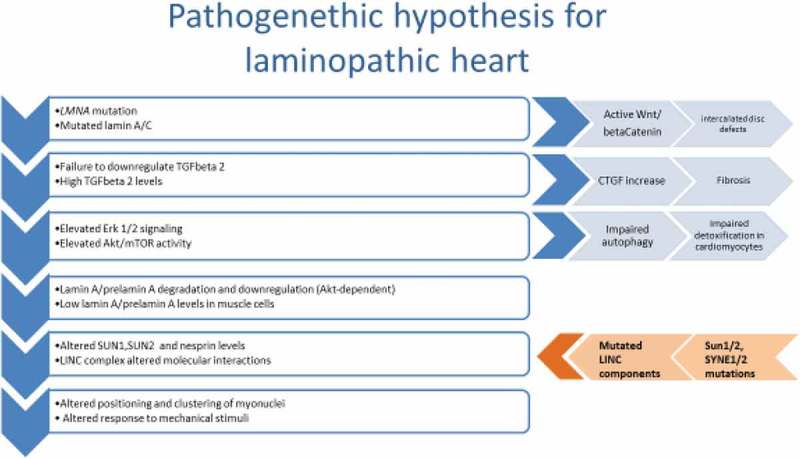
Main pathogenetic pathways in the pathophysiology of cardiolaminopathies.

The pathophysiology of cardiolaminopathies is complex, as summarized in Figure 2.

Two main pathogenetic pathways have been identified as the basis of cardiolaminopathies.

The first and better described pathway starts from activation of Erk 1/2 signaling leading to a profibrotic process, which encompasses TGFbeta 2-dependent activation of connective tissue growth factor [7,8]. The pathogenicity of Erk 1/2 activation has been proven in preclinical models. Inhibitors of Erk 1/2 or MEK or other molecules in the signaling pathway have been able to delay the onset of cardiomyopathy in *Lmna^H222P/H222P^* mice, which die prematurely due to cardiac impairment [9]. Further, activation of PI3-kinase leading to Akt/mTOR phosphorylation, has been demonstrated in laminopathic mice. The event downstream of mTOR activation was inhibition of autophagic detoxification in cells, a deleterious effect that could be counteracted in vivo by rapamycin or its analogs, mTOR inhibitors used in several clinical applications [10,11].

However, at least one additional pathomechanism is expected to play a role in laminopathic heart. Lamins are major sensors at the nuclear envelope and respond to both mechanical and chemical stimuli. The connection with extranuclear signal transducers is ensured by the Linker of Nucleoskeleton and Cytoskeleton (LINC) complex, a protein chain including integral proteins of the inner and outer nuclear membrane, such as SUN1/2 and nesprin1/2 (encoded by *SYNE1/2* genes), attached to lamins on the nuclear side and to actin and other cytoskeleton constituents on the cytoplasmic side. LINC proteins are reduced [12,13] or even mutated [13,14] in patients affected by cardiolaminopathies. The proven effect of LINC disorganization and reduced interplay between LINC components and lamins in laminopathic muscle cells is defective myonuclear positioning [12,14], which is a cause of altered muscle cell functionality [14]. These findings point to altered mechanosignalling transduction as a further pathogenetic event leading to heart dysfunction in laminopathies. Mechanosignalling is particularly relevant in conduction tissue, which deserves better investigation. In this respect, it is worth mentioning that reduced connexin 43 amount at the intercalated disks and altered disk morphology are linked to defects in Wnt/betaCatenin signaling in laminopathic heart [8].

These pathogenetic pathways are not mutually exclusive; instead they could be recapitulated in a unique mechanism that, starting from failure of mutated lamin A to regulate TGFbeta 2 levels [15], implies TGFbeta 2 increase [16] and TGFbeta2-dependent activation of Erk 1/2 and mTOR leading to fibrosis [7,15] and loss of autophagic detoxification [11]. In this context, elevated TGFbeta 2 and Akt/mTOR activation could also be the cause of reduced lamin A and prelamin A levels [15,17] at the cardiomyocyte nuclear envelope, a condition that favors LINC disorganization [12]. In agreement with this pathogenetic hypothesis, Erk 1/2 activation has been demonstrated in the presence of pathogenetic *LMNA* and *SYNE1* mutations [7,13], and altered positioning of muscle nuclei has been demonstrated in *LMNA, SYNE1* and *SUN1*-linked Emery-Dreifuss muscular dystrophy with cardiomyopathy [12–14,18].

Further studies will better define the complex molecular mechanism leading to cardiolaminopathies and confirm Erk ½ [9], mTOR [11], TGFbeta 2 [16] betaCatenin [8] or connective tissue growth factor [7] as targets of therapeutic intervention.

## Genotype-phenotype correlations

A series of studies have shown that there are no clear genotype-phenotype correlations, as different mutations in the same codon, or even the same mutation, may produce a variety of clinical symptoms [2,3]. This means that, despite common pathogenic pathways, the severity of the disease and the prevalent skeletal or cardiac muscle involvement might be regulated by modifying genes, as demonstrated in a family harboring *LMNA* and *TTN* mutations [18] or in families with *LMNA* and desmin gene mutations [19,20].

## Cardiac manifestations of laminopathies and clinical decision-making

The most common clinical manifestations are dizziness, syncope, palpitations, or even ischemic stroke due to cardioembolism (in the case of atrial fibrillation or atrial standstill) but also ventricular tachyarrhythmias and sudden death and/or worsening heart failure may occur, the latter usually at a later stage in the course of the disease [4,21–24]. We will describe the clinical characteristics of these symptoms, focusing on the need for a disease-oriented decision-making. As a matter of fact, the tendency of the disease to progress to more advanced stages with risk of life-threatening events, makes it necessary to interpret clinical findings and take appropriate decisions in the presence of clinical elements that in the context of this disease have a special relevance, differently from the significance that the same findings may have in the absence of a cardiolaminopathy.

Implantation of a pacemaker has been traditionally proposed for protecting against the consequences of bradyarrhythmias, but consideration for an implantable cardioverter defibrillator (ICD), able to interrupt malignant ventricular tachyarrhythmias, thus preventing sudden cardiac death, may become a priority in many patients who have the indication to implant a cardiac electrical device. Biventricular pacing is a form of cardiac stimulation, referred to as cardiac resynchronization therapy (CRT), that may improve cardiac function in the case of heart failure, low ejection fraction and ventricular dyssynchrony [25].

## Bradyarrhythmias, atrial fibrillation and atrial flutter

Bradyarrhythmias and supraventricular tachyarrhythmias are common features in laminopathies and often anticipate by decades the development of heart failure due to dilated cardiomyopathy [4,24]. Supraventricular arrhythmias include atrial flutter, atrial fibrillation, and atrial tachycardia, and may be associated with sinus node dysfunction (i.e. tachycardia-bradycardia syndrome). Atrial bradycardia at a young age (early second decade to fourth decade), followed by evolution to atrial standstill and with associated high-grade atrioventricular block or complete atrio-ventricular block are typical features of X-linked Emery-Dreifuss muscular dystrophy and pacemaker implant may be necessary even on an emergency basis, for syncope or low cardiac output, at an age around 20 [4], Table 1 shows the results of a complete review of the literature, with data on the specific arrhythmic profile of patients with various forms of laminopathies, according to genetic characterization.

**Table 1.** Patient characteristics in clinical studies on cardiac laminopathies, with data on arrhythmic profile, heart failure and stroke or thromboembolism.

Author, year	N of Patients and genetics	Age (yrs) and gender	Follow-up	% with AF, atrial flutter or atrial standstill; AV block or conduction disorders; VT or VF	% with heart failure (NYHA ≥2) and/or LV dysfunction; HTx or end stage HF; mean LVEF	% with stroke or thromboembolism
Becane H.-M [47], 2000	54 living relatives	N/A	N/A	AF, atrial flutter or atrial standstill 7% AV block or conduction disorders 20%, VT 9%	13%; HTx or end-stage HF 6%	N/A
Boriani G [4], 2003	18 patients with genetically confirmed X-linked (n 10, including 3 carriers) or autosomal dominant (n 8) EDMD	42.8 ± 19.6 M 72%	ranging from 1 to 30 y	Atrial fibrillation/flutter 61% with atrial standstill occurring in 5 of 11 (45%); AV block or conduction disorders 77.7%	HF 22.2%; HTx or end-stage HF 6%	36%
Sanna T [51], 2003	10 patients with EDMD and a LMNA gene mutation	25.6 ± 12.1 M 90%	median of 29 m	AF, atrial flutter or atrial standstill 70%; AV block or conduction disorders 60%; VT 50%	Mean LVEF 63.3 ± 8.5 %	N/A
Van Berlo J.H [23], 2005	299 carriers of lamin A/C mutations	31.2 M 55%	N/A	AF, atrial flutter or atrial standstill 16%; AV block or conduction disorders 45%	26%; HTx or end-stage HF 12%	N/A
Meune C [52], 2006	19	41.7 ± 13.4 M 74%	33.9 ± 21.0 m	AF, atrial flutter or atrial standstill 70%; AV block or conduction disorders 60%; VT 50%	Mean LVEF 58 ± 12 %	N/A
Van Tintelen J.P [43], 2007	61	N/A	N/A	AF, atrial flutter or atrial standstill 16%; AV block or conduction disorders 14%; VT 2%	HTx 11.5%	8%
Pasotti M [6], 2008	94 patients with LMNA gene mutations: 60 (64%) phenotypically affected 34 (36%) genotypically affected	Affected 40 ± 10 Healthy 23 ± 14 M 59%	median of 57 m	AF, atrial flutter or atrial standstill 25%; AV block or conduction disorders 85%; VT 13%	LVEF <35%: 35%; HTx 3%	N/A
van Rijsingen I.A [53], 2012	269 LMNA mutation +	36 (27–45) M 55%	median of 43 m	AF, atrial flutter or atrial standstill 36%; AV block or conduction disorders 47%; Sinus node dysfunction 13%; Malignant ventricular arrhythmias 18%	LVEF <45%: 37%; Mean LVEF: 55 % (35–63)	N/A
Saj M [54], 2012	103	59.7 ± 11.4 M 80.5%	N/A	AF, atrial flutter or atrial standstill 100%; AV block or conduction disorders 48.5%	LVEF <40%: 6.8%; Mean LVEF 55.6 ± 9.6%	Stroke 9.7%; TIA 2.9%
Anselme F [55], 2013	47	38 ± 11 M 55%	median of 95 m	AF, atrial flutter or atrial standstill 26%; AV block or conduction disorders 45%; VT 66%	LVEF <45%: 13%; HTx 19%	N/A
Maggi L [56], 2014	108 patients: 78 myopathic patients LMNA+ and 30 familial cases LMNA+ without myopathy	M 48%	7.5 ± 7 y	AF 43.5% (myopathic patients LMNA+)	78 myopathic patients LMNA +: 47.9% DCM, 1.4% HCM, 5.6% other CMP; HTx 10.3%	N/A
Forleo C [57], 2015	20 LMNA mutation-positive subjects (n = 10) LMNA mutation-negative subjects (n = 10)	36 ± 16 M 45%	N/A	AF, atrial flutter or atrial standstill 10%; AV block or conduction disorders 35%; VT 80%	Mean LVEF 60 ± 7 % HTx 5%	N/A
Steckiewicz R [58], 2016	21 EDMD patients	29 ± 9 M 76%	11 ± 8 y	N/A	N/A	N/A
Olde Nordkamp L.R [59], 2016	4916 patients with inherited arrhythmia syndromes 162 (3.3%) with LMNA	M 50%	33 m	AF, atrial flutter or atrial standstill 71%	N/A	N/A
Kumar S [60], 2016	25 LMNA mutation +	55 ± 9 M 92%	median of 7 m	AF, atrial flutter or atrial standstill 12%	Mean LVEF 34 ± 12%; HTx or end stage HF 44%	N/A
Kumar S [24], 2016	122 (104 phenotypically affected)	41 ± 14 M 57%	median of 7 y	AF, atrial flutter or atrial standstill 62%; AV block or conduction disorders 63%; VT 43%	LVEF ≤ 50%: 47%; HTx 27%	8%
Ollila L. 27], 2017	27 LMNA mutation carriers	48 M 48.1%	N/A	AF, atrial flutter or atrial standstill 55.6%; AV block or conduction disorders 59.3%; VT 77.8%	N/A	14.8% overall (33.3% in cases with dilated cardiomyopathies)
Sedaghat-Hamedani F [44], 2017	95 patients with left ventricular non-compaction (68 patients and 27 affected relatives; familial LVNC = 23.5%)	NA	median of 61 m	AF, atrial flutter or atrial standstill 29.4.%; VT 35.3%	Mean LVEF 38 ± 15.3%; HTx 10.3%	Stroke/TIA 8.8% Systemic thromboembolism 10.3%
Nishiuchi S [62]., 2017	77 LMNA mutation carriers (92% phenotypically affected)	45 ± 17 M 63.6%	median of 49 m	AF, atrial flutter or atrial standstill 58%; AV block or conduction disorders 81%; VT 26%	LVEF <50%: 45%; NYHA ≥3: 34%	N/A
Hasselberg N.E [61]., 2018	79 lamin A/C genotype +	42 ± 16 M 54%	7.8 ± 6.3 y	AF, atrial flutter or atrial standstill 61%; AV block or conduction disorders 65%; VT 60%	Mean LVEF 45 ± 13%; HTx 19%	N/A

Legend: AF: atrial fibrillation; AV: atrio-ventricular; EDMD: Emery-Dreifuss muscular dystrophy; HF: heart failure; HTx: heart transplantation; LMNA: lamin A/C; VT: ventricular tachycardia; LVEF: left ventricular ejection fraction; NYHA: New York Heart Association functional class; N/A: not available; m: months; Y: years; M: male; TIA: transient ischemic attack.

Low amplitude P wave, prolonged PR interval, left anterior hemiblock, and bundle branch block are typical features at the electrocardiogram of involvement of the conduction system and represent ‘red flags’ for the diagnosis of a cardiolaminopathy at a relatively young age.

In general, considering the high risk of bradyarrhythmias, the threshold for cardiac pacing through a device should be low and in the presence of atrio-ventricular conduction abnormalities or sinus node dysfunction disease-specific decision-making is recommended, in view of the natural history of the disease. In specific cases, the assessment of infra-hisian conduction at an electrophysiologic study (pathologic if measured HV interval is >70 ms) may help in decision-making with regard to the indication for permanent cardiac pacing.

**Figure 3.**
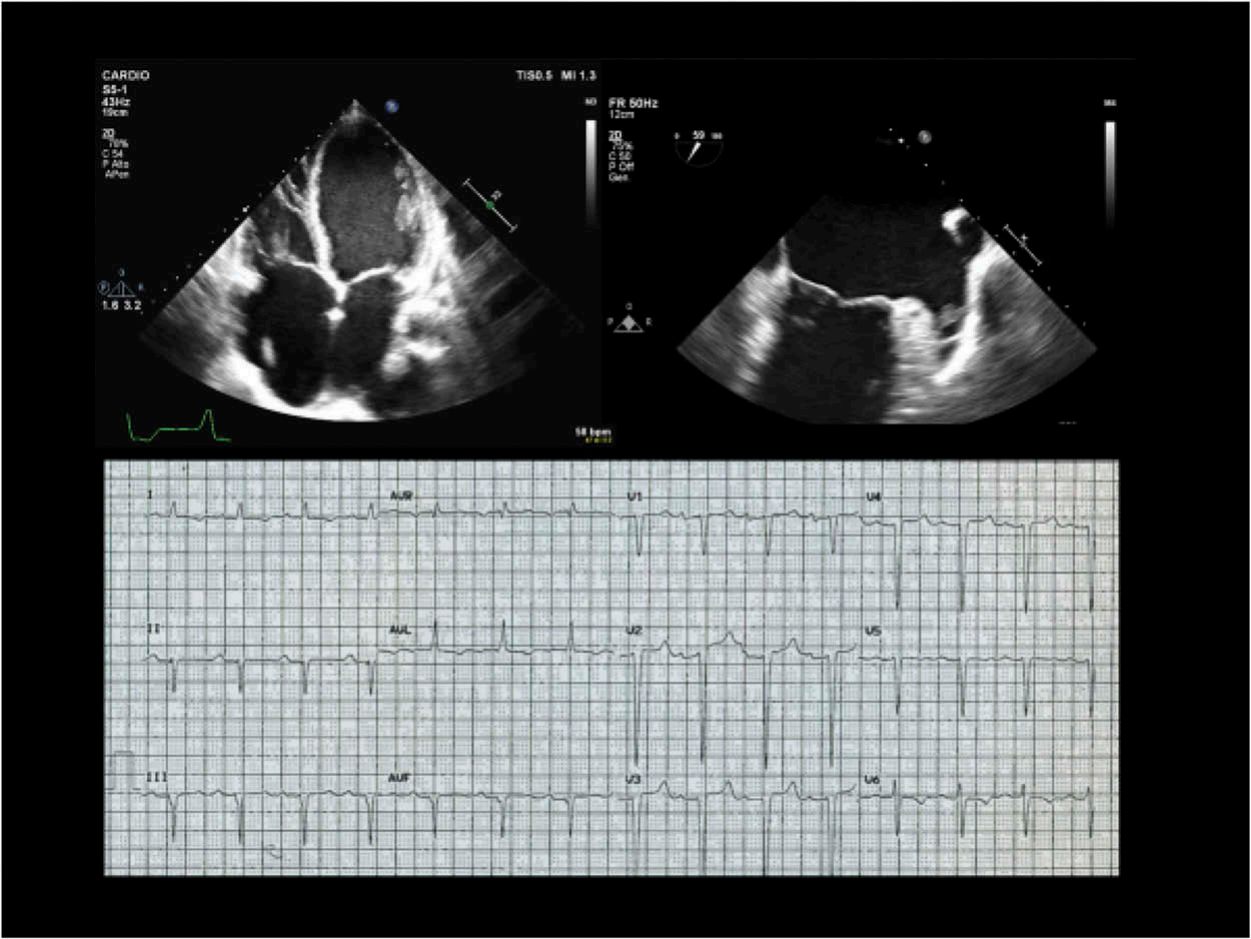
Echocardiographic (top) and electrocardiographic (bottom) findings of a typical cardiolaminopathy with paroxysmal atrial fibrillation. Panel A. Four-chamber view transthoracic echocardiography showing severe dilatation of both atria and severe dilatation and hypokinesia of both ventricles (ejection fraction of both left and right ventricles 20%). Panel B. Transesophageal echocardiography 60 degree view showing a trombus inside the left atrial appendage. Panel C. Electocardiogram: sinus rhythm, atrio-ventricular first degree A-V block, left anterior hemiblock and ventricular repolarization abnormalities.

Figure 3 shows an example of typical echocardiographic and electrocardiographic findings in a patient with cardiolaminopathy, who was in sinus rhythm, but with episodes of paroxysmal atrial fibrillation and presented an atrial thrombus in the left atrial appendage at transesophageal echocardiogram.

In the general population atrial fibrillation is usually an arrhythmia characteristic of the elderly [26], while it is rare at an age <30 and its onset at a young age should therefore raise the suspicion of a genetic cause and specifically of a laminopathy. Atrial fibrillation has been reported in around 60% of patients with a cardiolaminopathy [4,27], often with atrial fibrillation occurring at a young age (12–20 years) as the first cardiac manifestation of the disease, with or without neuromuscular symptoms [4]. In patients with cardiolaminopathy with the pattern of a dilated cardiomyopathy, the prevalence of atrial fibrillation is higher than 90% [27]. In the series reported by Olilla et al [27]., atrial fibrillation had a higher incidence in males as compared to female patients and men presented with atrial fibrillation at an earlier age (41 vs 54 years, p = 0.016).

The evolution of the pathologic processes at the atrial level is progressive and in a histopathologic post-mortem study on three hearts a marked loss of atrial myocardium was found, with evidence that the atrial myocardium had been replaced by adipose and fibrous tissue [28]. This impressive derangement of the atrial walls fits with the recent concepts of ‘atrial cardiomyopathy’[29], corresponding to any complex of structural, architectural, contractile or electrophysiological changes affecting the atria with the potential to produce clinically-relevant manifestations [30]. The extent and the progression of atrial structure derangement in cardiolaminopathies are the basis for the progression of clinical arrhythmias, with atrial fibrillation and flutter (which require some amount of excitable atrial tissue) typically followed over time by atrial standstill, as an expression of an extensive loss of viable atrial tissue [4].

The clinical implications of the pathological progression described at the atrial level is that catheter ablation procedures targeted to maintain sinus rhythm in patients presenting with atrial fibrillation, as well as repeated electrical cardioversions, have no chance of being successful at mid and long-term. Moreover, attempts to stimulate the atria by a dual-chamber pacemaker regularly fail due to progressive development of atrial standstill and atrial inexcitability by electrical stimuli, even if delivered at high electrical output [4].

## Thromboembolic complications and cardio-embolic stroke

Patients with laminopathies have a high risk of stroke due to cardioembolism, and this event, often disabling, has been reported to occur in up to 33–36% of cases in series of patients with cardiolaminopathies presenting atrial fibrillation in 56–61% of cases [4,27], (Table 1). In one series, ischemic stroke due to cardioembolism occurred in 36% of patients with atrial fibrillation (all not treated with oral anticoagulants), in half of the case aged <45, and resulted in significant disability.

**Figure 4.**
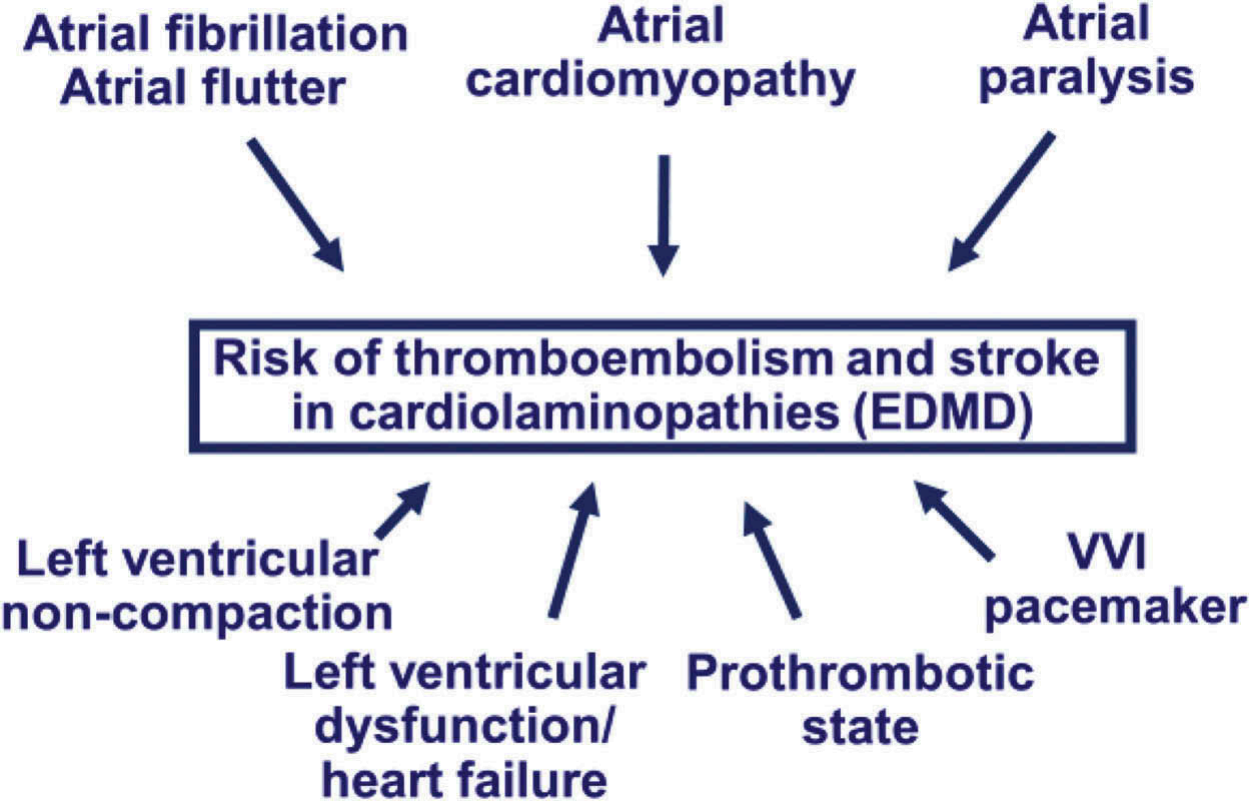
Factors affecting the risk of thromboembolism and stroke in cardiolaminopathies and Emery-Dreifuss muscular dystrophy. Legend: EDMD: Emery-Dreifuss muscular dystrophy.

It is well known that in patients with atrial fibrillation the risk of thromboembolism can be predicted by the CHA_2_DS_2_-VASc score, but this score has been validated in clinical contexts and patient ages completely different from laminopathies [31]. The association between cardiolaminopathies and a high risk of cardioembolic stroke even at a young age and with low CHA_2_DS_2_-VASc scores indicates that the conventional risk stratification adopted for atrial fibrillation patients cannot be translated to this specific setting as a guide to anticoagulation and that the high risk of cardioembolic stroke, dependent on many interrelated factors (Figure 4), suggests starting oral anticoagulation, in the absence of absolute contraindications, in any patient with a cardiolaminopathy and with documented atrial fibrillation or with other atrial tachyarrhythmias (atrial flutter, atrial tachycardia) or with atrial paralysis. Despite the absence of controlled trials, the high occurrence of ischemic stroke at young age in untreated patients suggests this disease-specific recommendation [4,27]. The current availability of non-vitamin K antagonist anticoagulants for so-called non-valvular atrial fibrillation [32], that do not require periodic monitoring of the level of anticoagulation, greatly facilitates the initiation of this antithromboembolic prophylaxis [33–35].

Atrial fibrillation may be asymptomatic or associated with atypical symptoms in 30–40% of unselected patients [36], and the implications for outcome are poor, since the risks of cardiovascular death, all-cause death and stroke are higher in patients with asymptomatic versus symptomatic atrial fibrillation [37,38]. No specific data are available on the ratio of asymptomatic versus symptomatic atrial fibrillation in cardiolaminopathies, but the frequent association with alterations of atrio-ventricular conduction, limiting the occurrence of fast ventricular rates, makes the chance of asymptomatic atrial fibrillation even higher than in the general population. In view of the risk of stroke and thromboembolism related to atrial fibrillation, regardless of the presence/absence of symptoms like palpitations, periodic checks with a 12-lead electrocardiogram are indicated in patients with known cardiolaminopathy or in lamin A/C mutation carriers. The role of opportunistic or systematic screening, using hand-held devices for single-lead electrocardiogram or ECG event monitors or using more recent user-friendly tools, such as smartphones or bracelets with dedicated software, has still to be assessed in this specific context [39,40] but may be of great value in view of the relatively high pre-test probability of developing atrial tachyarrhythmias. Similarly, the diagnostic capabilities of implanted devices for bradyarrhythmias and prevention of sudden cardiac death (i.e. pacemakers and defibrillators) can help in detecting atrial fibrillation through their diagnostic capabilities, even if the implant of devices without an atrial lead limits the diagnostic accuracy unless dedicated algorithms are implemented [41] .

In a study comparing lamin A/C mutation carriers [42] (atrial tachyarrhythmias in 63% and depressed systolic function in 46% of cases) with patients with dilated cardiomyopathy not due to lamin A/C mutations, the prevalence of thromboembolic complications (more commonly arterial, but also venous) was higher during follow up in the cohort of lamin A/C mutation carriers and after adjustment for possible confounders, including atrial tachyarrhythmias and left ventricular ejection fraction, lamin A/C mutation carriership was independently associated with an increased risk of thromboembolic complications (HR 4.8, 95% CI: 2.2–10.6). The same study [42] found a higher thrombin generation in lamin A/C carriers and an altered platelet function suggesting an associated prothrombotic state that may explain the previous findings, as well as the observations of van Tintelen et al. [43] of a high risk of thromboembolism in specific families, regardless of identifiable risk factors for thromboembolism and of atrial tachyarrhythmias. Further research is needed in this field.

As shown in Figure 3, other factors, such as the use of VVI pacemakers and the frequent presence of left ventricular non-compaction [44], may be additional aspects increasing the risk of thromboembolism and stroke.

## Dilated cardiomyopathy

The phenotype of dilated cardiomyopathy, characterized by left ventricular or biventricular dilatation, reduced systolic function and progressive heart failure, is, in general, genetically heterogeneous, dependant on genes encoding cytoskeletal, sarcomeric, mitochondrial, desmosomal, nuclear membrane, and RNA-binding proteins [45]. Lamin A/C mutations are found in around 5% of patients with non-familial idiopathic dilated cardiomyopathy, in 5%–10% of patients with idiopathic familial dilated cardiomyopathy and in 33% of cases of familial dilated cardiomyopathy with conduction defects [3,45].

From the clinical point of view, the pattern of dilated cardiomyopathy related to the *LMNA* gene does not present unique characteristics able to clearly differentiate it from dilated cardiomyopathy related to other genes or dilated cardiomyopathy of non-genetic origin, although the finding of muscular involvement (even if CPK is elevated in only one third of cases), atrio-ventricular blocks and atrial fibrillation or atrial flutter are considered as ‘red flags’ predicting a much higher chance of a lamin A/C mutation [46–48]. In Table 1 data from literature on dilated cardiomyopathies and heart failure in reported series of patients with cardiolaminopathies are reported. Left ventricular dilatation may be of variable degree, also with the pattern of ‘mildly dilated cardiomyopathy’ and can usually be detected by two-dimensional echocardiography and/or with cardiac magnetic resonance imaging (MRI). The systolic function, as measured by left ventricular ejection fraction, has to be measured (values below 50% characterize systolic dysfunction) and monitored over time in order to assess disease progression. The expression of the pattern of dilated cardiomyopathy was found to be age-dependent, with development of the phenotype between 20 and 39 years in two thirds of the cases and complete penetrance by 60 years [6].

Patients can be diagnosed as being affected by a dilated cardiomyopathy as the expression of a cardiolaminopathy following a cardiological workup performed for symptoms of heart failure or for arrhythmic events or can be diagnosed incidentally or during family screening. Usually, the expression of a pattern of dilated cardiomyopathy follows that of the arrhythmic manifestations, or the two manifestations can be associated at the time of the first diagnosis [6]. Dilated cardiomyopathy related to laminopathies can present an aggressive and rapid evolution to very severe and potentially life-threatening conditions, with a worse natural history in comparison with other forms of non-ischemic dilated cardiomyopathy [6,21]. In these cases of progressive and worsening heart failure, other options in addition to pharmacological treatment can be considered. Appropriately selected patients may benefit from cardiac resynchronization therapy and even heart transplantation or mechanical circulatory support may be needed in selected patients [3].

Patients with cardiolaminopathy with the phenotype of dilated cardiomyopathy often present left ventricular non-compaction, i.e. excessive left ventricular trabeculae, deep intratrabecular recesses, and a thin compacted myocardial layer [44]. This finding, detectable at 2-dimensional echocardiography or magnetic resonance imaging is not unique to cardiolaminopathies, but lamin A/C mutations are among the most frequent genetic abnormalities detectable in patients with non-compacted myocardium [44].

In an observational study of 94 patients with dilated cardiomyopathy due to lamin A/C mutations, the outcome in years was predicted at multivariable analysis by a series of variables, with New York Heart Association functional class III to IV and history of highly dynamic competitive sports for ≥10 years being independent predictors of total events (arrhythmias, heart failure, death) and splice site mutations and competitive sport being associated with increased risk of sudden cardiac death [6].

In the series of cases recently reported by Kumar et al [24] during a median follow up of 7 years, 24% of patients without left ventricular dysfunction at baseline developed new left ventricular dysfunction and 7% developed end-stage heart failure. Left ventricular dysfunction (ejection fraction <50%) was associated with end-stage heart failure or death, but the mode of presentation (with isolated or combination of clinical features) did not predict end-stage heart failure or death.

Cardiac resynchronisation therapy (CRT) is an electrical therapy for heart failure that can be applied in cases with electrical dyssynchrony (bundle branch block or wide QRS at the ECG) and has been shown to improve left ventricular systolic performance, morbidity and mortality in patients with heart failure [49,50]. The beneﬁcial effects of CRT on LV reverse remodeling and systolic function are very clear in dilated cardiomyopathy with LV non compaction, a setting where a high proportion of patients may exhibit a so called ‘super-response’, with the evidence that the greater the area of noncompaction (higher number of non-compacted segments) the greater the chance of achieving a favourable CRT response and greater LV reverse remodeling [25].

## Ventricular tachyarrhythmias and sudden cardiac death

Sudden cardiac death due to ventricular tachyarrhythmias constitutes the most dramatic and devastating event that may occur in cardiolaminopathies. Sudden cardiac death can be the first manifestation of the disorder or may occur during the course of the disease.

A detailed review of all the literature published on the risk of ventricular tachyarrhythmias, and on device activations in carriers of an implantable cardioverter defibrillators (ICDs) are shown in Table 2, in relationship to the series of patients with laminopathies reported in Table 1 [4,6,23,24,27,43,44,47,51–62]. In the largest observational study, reported by Kumar et al [24], the cumulative occurrence of sustained ventricular tachyarrhythmias was 34% at 7 years, and among patients with no history of ventricular tachyarrhythmias the incidence was 22% during a 7-year follow-up. In the same series, appropriate ICD activations occurred in 50% of patients with an ICD implanted for primary prevention of sudden cardiac death, with a rate of appropriate interventions (3% to 7%/year) comparable to appropriate ICD interventions occurring in high-risk patients with idiopathic or post-infarction dilated cardiomyopathy. In this series of patients, who were implanted with a defibrillator in 59% of cases, male sex, non-missense mutations, and left ventricular dysfunction were associated with development of sustained ventricular tachyarrhythmias, while no relationship was found with the mode of presentation (with isolated or combination of clinical features) [24]. These findings are in line with a previous multicentre study, where a series of risk factors emerged as predictors of the occurrence of ventricular tachyarrhythmias (male gender, non- sustained ventricular tachycardia, left ventricular ejection fraction <45% and non-missense mutation), thus providing an important clinical guide for the decision to implant an ICD [53] (Table 2) [4,6,23,24,27,43,44,47,51–62]

**Table 2.** Type of cardiac electrical devices implanted for arrhythmic problems, device-related complications, ICD shocks and outcome, in terms of sudden cardiac death, stroke and all-cause mortality in clinical studies on cardiac laminopathies.

Author, year	% PM or ICD	ICD/PM complications	ICD appropriate shocks	SCD	All-cause mortality
Becane H.-M [47], 2000	PM 11%	N/A	N/A	N/A	N/A
Boriani G [4], 2003	PM 56%	PM complications in 30%	N/A	N/A	11%
Sanna T [51], 2003	PM 40%	N/A	N/A	10%	N/A
Van Berlo J.H [23], 2005	PM 25%	N/A	N/A	46%	25%
Meune C [52], 2006	ICD 100%	Inappropriate ICD shocks in 5%	42%	N/A	N/A
Van Tintelen J.P [43], 2007	PM 8% ICD 1.6%	N/A	NA	13%	NA
Pasotti M [6], 2008	PM 21% ICD 17%	N/A	12%	16%	N/A
van Rijsingen I.A [53], 2012	ICD 40%	Inappropriate ICD shocks in 9%	24%	5%	17%
Saj M [54], 2012	20.3% PM 1.9% CRT-D	N/A	N/A	N/A	N/A
Anselme F [55], 2013	26% PM 45% ICD	ICD lead fracture in 14%	52%	N/A	15%
Maggi L,[56] 2014	PM or ICD 44%	N/A	N/A	N/A	12.8%
Forleo C [57], 2015	PM 5% ICD 25%	N/A	N/A	N/A	N/A
Steckiewicz R [58], 2016	PM 95% ICD 5%	N/A	N/A	N/A	N/A
Olde Nordkamp L.R [59], 2016	ICD 100%	Inappropriate ICD shocks in 13 %; ICD-related complications in 25 %	N/A	N/A	Cardiac mortality 3.1%
Kumar S [60], 2016	PM 8% ICD 40% CRT-D 36%	N/A	Median of 1.5 shocks (Q25–Q75, 0–9) shocks in the month before ablation Median of 0 shocks (Q25–Q75, 0–1) in the 6 months after ablation.	4%	Cumulative estimate for all-cause mortality 26 ± 11%
Kumar S [24], 2016	ICD 59%	N/A	N/A	3%	22%
Ollila L [27], 2017	ICD 33.3%	N/A	N/A	N/A	N/A
Sedaghat-Hamedani F [44], 2017	ICD 32.3%	N/A	18.2%	N/A	13.2%
Nishiuchi S [62], 2017	PM 21% ICD 43%	N/A	24%	3%	N/A
Hasselberg N.E [61], 2018	ICD 62%	N/A	28%	N/A	8%

Legend: CRT_D: cardiac resynchronization therapy with defibrillation back-up; ICD: implantable cardioverter-defibrillator; PM: pacemaker/A: not available.

## Remote monitoring

**Figure 5.**
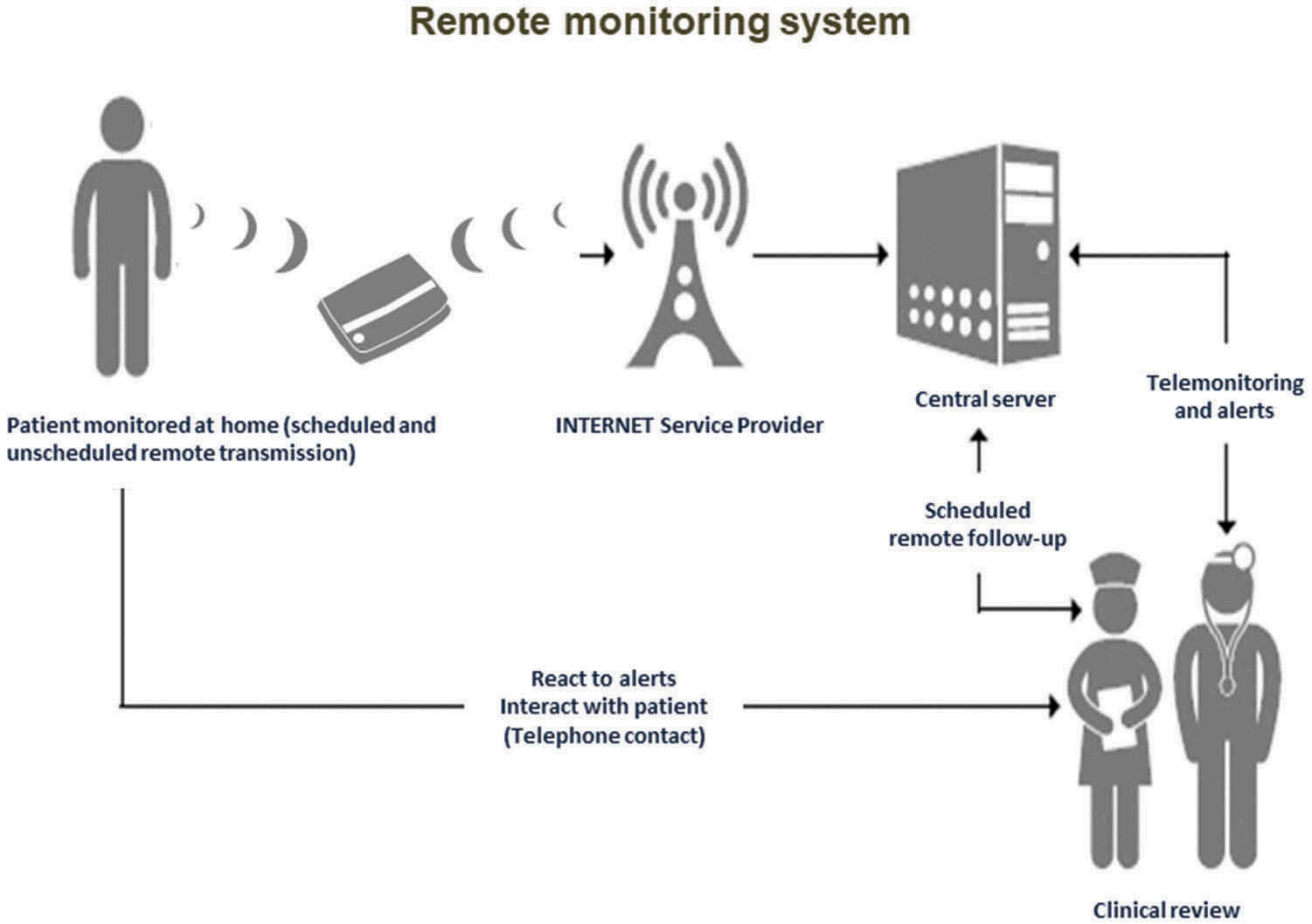
Remote monitoring of patients with an implanted device: the flow of information is characterized by transmission to physicians and nurses for appropriate decision-making on actionable clinical events.

Thanks to technological improvement, data on electrical device parameters can be remotely transmitted and this may make it possible to avoid conventional in-hospital device checks. However, the added value of this technology is the possibility to monitor the patient’s clinical status and to take appropriate clinical decisions on the basis of detected events (e.g. new arrhythmias) or signs of worsening clinical conditions, specifically worsening heart failure. Figure 5 shows the flow of information in a remote monitoring system of patients with an implanted device and transmission of device-detected events to physicians and nurses for appropriate decision-making. The evolving capabilities of implanted devices to monitor the patient’s status may imply a shift from device-centered follow-up to patient-centered follow-up [63]. In fact, telecardiology and remote control make it possible not only to evaluate the device parameters but also to detect arrhythmias and signs of worsening heart failure with a potentially important impact on clinical and economical outcomes [64]. In a group of patients with neuromuscular disorders and with a high risk of clinical events, remote monitoring could represent an important clinical opportunity, whose advantages are in addition to avoidance of the inconvenience of in-hospital checks, particularly tiresome in patients with neuromuscular involvement conditioning functional impairment.

Several trials demonstrated that remote control is safe and reduces delay in detection of events such as atrial fibrillation and worsening heart failure [63,65,66]. The MORE CARE randomized trial revealed a median 27-day reduction in delay from actionable event detection to medical decision in the device group with remote control compared to a control group without remote data transmission [63]. The IN-TIME randomized trial recently demonstrated that automatic, daily, multiparameter telemonitoring is associated with improved clinical outcome for patients with heart failure, with a significant reduction in all-cause mortality [67]. This observation requires confirmation in additional controlled trials.

## The future of electrical therapies in laminopathies

New technologies introduced in the last 5 years can help physicians to reduce device-related complications. An important innovation is the extension of device longevity to up to 10 years. through improvement of device batteries [68,69] This makes it possible to reduce the number of device replacements with benefits related to reduced risk of infections and reduced long-term costs [69].

Despite great technical advances over the last decades, device implants that include transvenous leads are still associated with some risk of complications ascribed to the damage that mechanical stress can induce in the leads (lead fracture, lead erosion). A leadless pacemaker is now available for treating bradyarrhythmias, thus avoiding the risk of lead-related complications. While such risks are statistically rare, they represent an economic burden to hospitals and the healthcare system because of the large number of pacemaker patients (over 4 million worldwide). In the Follow Pace study, the rate of complications was 5.5% in the 6-year follow up of an unselected population of pacemaker patients [70]. These data are confirmed by the Danish registry that reported a complication rate of 7% in around 6000 patients with a pacemaker [71]. The rates of the specific types of device complications are: lead-related in 2–5%, pocket-related in 1–4%, infection-related in 1–2% and pneumothorax in 1–2% [70,71]. The option of leadless pacemakers may minimize the risk of these complications. At present, only single chamber pacemakers are available, but this could represent a suitable option for patients with atrial fibrillation or atrial standstill and neuromuscular disorder. The early experience with a leadless pacemaker reported a 99% implant success rate, with 96% of patients free from major complication [72]. In a comparison between historical controls and leadless pacing implants, the relative risk of major complications was reduced by 48% with the leadless technology [73]. The data of the ‘real world’ MICRA leadless pacemaker have recently confirmed the short-term reliability of this type of device implant [74,75]. The major drawback of this technology is the limitation of single-chamber pacing, as well as the high cost. The single chamber pacemaker is a device that can be proposed for 15–20% % of patients with bradyarrhythmias [72,74]. In patients with neuromuscular disorder (such as Emery-Dreifuss muscular dystrophy) with atrial fibrillation or atrial standstill, who do not present indications for a defibrillator, implant of a leadless pacemaker could represent an emerging option in appropriately selected cases. In the future the possibility to combine a leadless pacemaker with a subcutaneous ICD may allow to upgrade device therapy in case of evolution of the disease, with left ventricular dysfunction developing during follow up and suggesting over time the need for defibrillation back-up, while avoiding intravascular leads.

**Figure 6.**
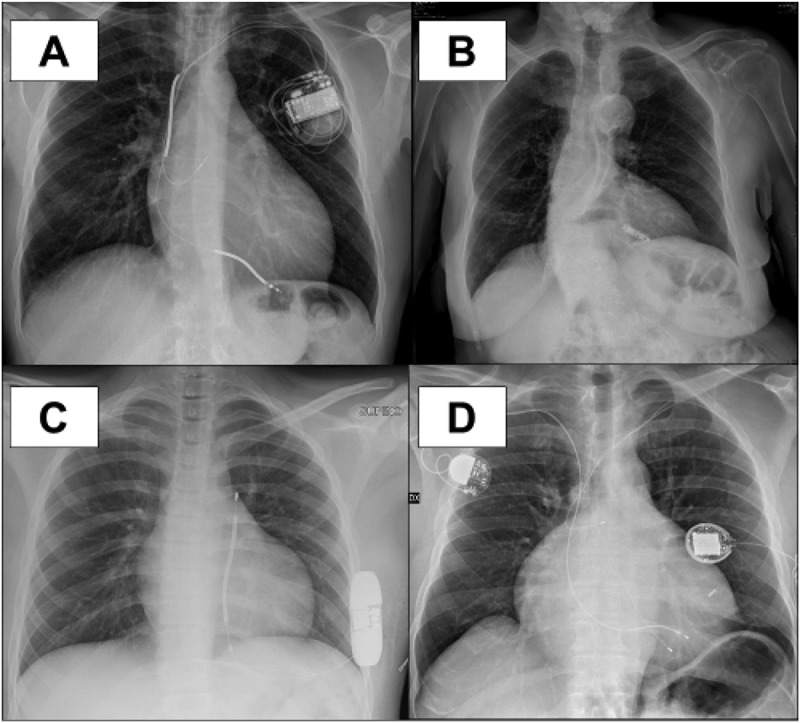
Chest X rays showing different types of implanted electrical devices that can be considered as current therapeutic options, according to the indications of consensus guidelines. A: transvenous cardioverter-defibrillator for prevention of sudden arrhythmic death; B: leadless VVI pacemaker; C: subcutaneous implantable cardioverter defibrillator; D: leadless ultrasound-based left ventricular pacing for cardiac resynchronization therapy in combination with a conventional pacemaker positioned in the right infraclavicular region.

As reported before, cardiac resynchronization therapy, which is an effective treatment for heart failure patients with left ventricular dysfunction and wide QRS at the ECG [46], is based on biventricular pacing, and in patients with sinus rhythm requires an atrial lead, a right ventricular lead and a left ventricular lead, thus increasing the risk of lead complications over time. An innovative system for cardiac resynchronization therapy has been developed, including left ventricular leadless pacing through a wireless, ultrasound-based system and 2 subcutaneous components (battery and transmitter) in conjunction with a co-implanted right ventricular pacing system (Figure 6 panel D). This system is costly and is currently proposed for patients with a failed or ineffective conventional transvenous resynchronization implant, a setting including around 15% of candidates for cardiac resynchronization therapy. The advantage of this kind of leadless pacemaker is that a complete endothelization of the leadless pacemaker was documented 3 weeks after the implant. In a preliminary study, the implant success rate was high (97%) but the rate of complications was 8.6%, thus placing this alternative only as second or third (after the surgical approach) option. A specific advantage of left ventricular endocardial pacing is the opportunity to pace the ventricular target site without phrenic nerve stimulation, usually with very good pacing threshold.

The subcutaneous ICD is a new effective therapy for ventricular tachyarrhythmia. Data show that there are no differences in overall complications between subcutaneous and transvenous ICD but the complications of subcutaneous ICDs can be resolved easier [76]. The rate of lead complications is lower in subcutaneous ICDs, but non-lead-related complication are more common. Infections, erosions, defibrillation failure and inappropriate shocks have been reported to occur more frequently in subcutaneous ICDs [76]. A recent metanalysis confirmed these data [77], with the exception of inappropriate shocks due to T wave oversensing which were observed more frequently in patients with a subcutaneous ICD as compared to transvenous ICDs [77]. A new algorithm is available in subcutaneous ICDs for reducing T wave oversensing. Subcutaneous ICDs are currently not able to pace the ventricles and therefore cannot be used in case of bradyarrhythmias. The surgical procedure is a little more complex than a conventional transvenous implant. Therefore, in the specific setting of cardiolaminopathies implant of a subcutaneous ICD can be taken into consideration only when there is no indication for CRT [78], significant bradyarrhythmias have been excluded, and the avoidance of intravascular leads is a clinical priority (e.g. previous device system infection).

Figure 6 illustrates cases of clinical use of different types of new devices for electrical therapies of the heart that are already available for appropriately and carefully selected patients.

## Conclusions

Lamin A/C gene mutations can be associated with myocardial diseases, usually referred to as ‘cardiolaminopathies’ characterized by arrhythmic disorders and/or left ventricular or biventricular dysfunction up to an overt picture of heart failure. The phenotypic cardiac manifestations of laminopathies are frequently mixed in complex clinical patterns and specifically may include bradyarrhythmias (sinus node disease or atrioventricular blocks), atrial arrhythmias (atrial fibrillation, atrial flutter, atrial standstill), ventricular tachyarrhythmias and heart failure of variable degrees of severity. Family history, physical examination, laboratory findings (specifically serum creatine phosphokinase values) and ECG findings are often important ‘red flags’ in diagnosing a ‘cardiolaminopathy’. *S*udden arrhythmic death, thromboembolic events or stroke and severe heart failure requiring heart transplantation are the most dramatic complications of the evolution of cardiolaminopathies and appropriate risk stratification is clinically needed, combined with clinical follow-up.

Treatment with cardiac electrical implantable devices is indicated in case of bradyarrhythmias (implant of a device with pacemaker functions), risk of life-threatening ventricular tachyarrhythmias (implant of an ICD) or in case of heart failure with wide QRS interval (implant of a device for cardiac resynchronization).

New technologies introduced in the last 5 years can help physicians to reduce device-related complications, thanks to the extension of device longevity and availability of leadless pacemakers or defibrillators, to be implanted in appropriately selected patients, after careful and personalized considerations. The indications to implant a specific type of device (a pacemaker, an ICD or a device for CRT) has to consider the current status of the disease, its potential progression/evolution with the associated risk of developing heart failure and/or life-threatening ventricular arrhythmias, as well as the risk of implant-related complications. In a multiform spectrum of diseases, such as cardiolaminopathies, with variable phenotypic expressions and with a potential evolution to most severe stages that remains of difficult prediction in individual cases, the process of clinical decision-making with selection of a specific type implantable electronic devices has many gray zones and is not deeply developed in the recommendations of consensus guidelines for device implant, which are typically referred to single diseases (atrial fibrillation, heart failure, etc.) with minimal reference to the complex pattern of cardiolaminopathies. A personalization of decision making is absolutely needed in patients with cardiolaminopathies, taking into account discussion of specific cases in a multidisciplinary team. The physician-patient interaction is also of primary importance, with involvement of relatives when appropriate, and a detailed description of the specificities of implanted devices is needed for full acceptance of this type of therapy.

An improved knowledge of the complex pathophysiological pathways involved in cardiolaminopathies and in the determinants of their progression to more severe forms will help to improve clinical management and to better target pharmacological and non-pharmacological treatments.
